# Immunohistochemistry staining of Eag1 and p16/Ki-67 can help improve the management of patients with cervical intraepithelial Neoplasia after cold knife conversion

**DOI:** 10.1186/s13000-024-01523-z

**Published:** 2024-07-11

**Authors:** Shikang Qiu, Qiannan Wang, Huihui Jiang, Limin Feng

**Affiliations:** 1 Department of Gynecology, Beijing Tiantan Hospital, Capital Medical University, Beijing, 100070 China; 2 Department of Clinical Laboratory, Qingdao Central Hospital, University of Health and Rehabilitation Sciences (Qingdao Cancer Hospital), Qingdao, Shandong 266000 China

**Keywords:** Cervical intraepithelial neoplasia, Eag1, p16, Ki-67, Follow-up

## Abstract

**Background:**

Immunohistochemistry (IHC) is widely used in the management of patients with cervical intraepithelial neoplasia (CIN) but still has many limitations in clinical practice. We analyzed the correlation of new biomarkers with the severity of CIN and follow-up outcomes in patients after conization to improve the management of patients with CIN.

**Methods:**

IHC staining of Eag1 and p16/Ki-67 was performed on cervical tissue sections from 234 patients with suspected CIN2/3. After a series of follow-ups, including human papillomavirus (HPV) test and thinprep cytologic test (TCT) for 1–2 years, the outcomes were collected. IHC scores of biomarkers and follow-up results were used to analyze the correlation and assess the diagnostic efficiency of biomarkers.

**Results:**

The IHC staining intensity of Eag1 and p16/Ki-67 was significantly different from that of the CIN1-3 groups (*p* < 0.05). Eag1 expression scores were significantly different in the distribution between the two follow-up groups (*p* < 0.001). ROC curves based on the correlations between the follow-up outcomes and the Eag1 scores and IS of p16/ki-67 showed that Eag1 had a greater AUC (0.767 vs. 0.666). Logistic regression analysis of the combination of biomarkers revealed a greater AUC value than any single biomarker.

**Conclusions:**

Eag1 expression was significantly correlated with CIN grade and follow-up outcomes after conization. IHC staining of combinations of biomarkers of Eag1, p16 and Ki-67 may help us to improve the ability to identify risk groups with abnormal follow-up outcomes after treatment for CIN.

## Introduction

Cervical cancer (CC), the second most common malignant tumor in women worldwide, emerges from cervical intraepithelial neoplasia (CIN) and is closely related to persistent high-risk human papillomavirus (HR-HPV) infection [1]. Advanced CC has a high mortality rate [2]. However, CIN can be effectively tested and treated [3]. The clinical consensus points out that patients with different grades of CIN need different management strategies, including follow-up or surgical resection [4]. Histological diagnosis based on HE staining is considered the “gold standard”, but some CINs are difficult to distinguish due to its subjective nature [5]. In addition, the clinical consensus believes that continuous follow-up for patients with CIN after treatment is very important. Researches have indicated that even among patients undergoing cervical surgery, the persistence or recurrence of CIN occurs in 5-35% of patients after treatment, and they often have human papillomavirus (HPV) test and thinprep cytologic test (TCT) abnormalities meanwhile [6]. Therefore, researchers hope to find effective specific biomarkers to help grade CIN and predict the risk of CIN persisting after treatment. ASCCP guidelines in 2024 noted that p16/Ki-67 dual staining was significantly helpful in guiding the management of HPV-positive patients [7]. Zummeren et al. proposed a classification system called the immune score (IS) based on the cumulative score of p16 and Ki-67 expression, which has proven to be an effective integrated indicator [8]. However, some researchers, such as Miralpeix et al., also point out the deficiency of overdiagnosis of CIN1 [9]. There are conflicting views regarding the ability of p16/Ki67 to predict postoperative outcomes in patients following treatment. Wang et al. suggested that p16 and Ki67 are clinically significant for guiding disease progression and prognosis at follow-up [10]. However, Zhong et al. suggested that there was no significant correlation [11]. This suggests that it is necessary to search for new biomarkers related to CIN severity and postoperative follow-up.

The relationship between ion channels and cancer has been well established [12]. Ether-a`-go-go-1 (Eag1) has attracted the attention of cancer researchers due to its high expression in tumors and low expression in healthy tissues. Eag1 is a protein composed of 989 amino acids, which is encoded by the H member 1 (KCHN1) gene of the potassium voltage gated channel subfamily and is responsible for the selective transport of K^+^ [13]. Eag1 has a restricted distribution limited to the central nervous system. In contrast, it has also been demonstrated that it is expressed in more than 70% of various tumors and functions in the proliferation, survival, angiogenesis and invasion of cancer cells [14]. Studies have shown that the HPV E6 E7 protein can down-regulate miR-34a and up-regulate transcription factor E2F1 by interacting with the RB-P53 pathway in cervical cancer cells, which can increase the expression of Eag1 [15]. In addition, our previous published study confirmed that inhibiting the electrophysiological function of Eag1 can reduce the viability, migration and invasion of HeLa cells [16]. We hypothesized that Eag1 might be involved in the early stages of HPV infection in cervical cells and therefore could be used as an indicator of the biological characteristics of CIN.

In this study, immunohistochemistry (IHC) staining for Eag1 and p16/Ki67 was performed on cervical tissue samples from patients with CIN to generate an immune score. We focused on the correlation of Eag1 expression with the severity of CIN and the correlation between Eag1 and HPV/TCT test abnormalities in CIN patients after conization. We also aimed to combine the expression score of Eag1 and the IS of p16/Ki-67 to explore the ability of these biomarkers to predict prognosis at follow-up. Because studies have confirmed the value of HPV combined with cytology testing in follow-up after CIN treatment and abnormal test results indicate a high risk of persistence or recurrence of CIN, we set the end point as the presence of abnormal HPV and cytology tests within 1–2 years after cold knife conization (CKC) [17]. Our study would help to identify these “risk patients” with abnormal follow-up outcomes and focus on them in the follow-up in advance.

## Methods

### Samples

Patients with CIN2 and above diagnosed by colposcopy biopsy were randomly recruited from who underwent cervical cancer screening at the gynecological clinic of Beijing Tiantan Hospital, Capital Medical University from January 2020 to January 2023. All patients were hospitalized for CKC and followed up for 1–2 years with HPV and TCT tests every 3–6 months. Cervical specimens were preserved, and HE staining was performed to confirm the final pathological diagnosis. Patients with cancer and no detectable CIN were excluded, as well as pregnant and lactating women, patients with a history of other cervical or vaginal lesions, patients with other malignancies, and patients with other serious systemic diseases. Finally, 234 eligible patients were identified. All patients signed the informed consent form, and this study has been approved by the Ethics Committee of Beijing Tiantan Hospital, Capital Medical University (Ethics number: KY2021-168-02).

## Materials and methods

Tissue specimens obtained from biopsy or CKC were fixed in 10% formalin and embedded in paraffin. Serial sections with a thickness of 0.4 mm were selected from each sample for staining. According to the results of HE staining, the most severe and representative part of the samples from each patient was selected for analysis. All pathological results were obtained by two experienced pathologists.

### Immunohistochemistry and scoring of Eag1 and p16/Ki-67

All sections were stained with HE, Ki-67, and p16 immediately after surgery. In-between sections were used for immunostaining with monoclonal antibodies against Eag1 (1:500, NBP1-84935) after the pathological diagnosis results were confirmed. All IHC results were obtained on an automated Ventana staining machine (Ventana Benchmark ULTRA, Ventana Medical Systems, Roche, USA). The IHC scoring criteria were as follows: two pathologists who were blinded to the HPV and TCT results scored the expression of Ki-67 (score 0–3), p16 (score 0–3), and Eag1 (score 0–3) in the most dysplastic area. Briefly, with no increase in Ki-67 nuclear staining, only CIN cervical intraepithelial neoplasia staining of cells in the basal layer was scored as 0, and increased Ki-67 staining up to the lower one-third, two-thirds, or more than two-thirds of the epithelium was scored as 1, 2, or 3, respectively. Negative or patchy p16 staining was scored as 0, and diffuse staining up to the lower one-third, two-thirds, or more than two-thirds was scored as 1, 2, or 3, respectively. Using these scores, a cumulative IS of P16/Ki-67 (0–6) was generated for each biopsy sample [8]. Eag1 follows a similar approach. It was scored by distribution pattern in the squamous epithelium (basal one third, basal two third, full thickness, 0–3) for CINs.

### Statistical analysis

In the univariate analysis, chi-square test or Fisher’s exact test was used to analyze the categorical variables such as the IHC score and the severity and prognosis of CIN. The performance of the Eag1 score and IS of p16/Ki-67 was assessed using receiver operating characteristic (ROC) curves and calibration curves, with the area under the ROC curve (AUC) ranging from 0.5 to 1. Results with a p value < 0.05 were considered significant. All the statistical analyses were performed using R software (version 4.2.2), along with MSTATA software.

## Results

### Demographics

**Fig. 1:**
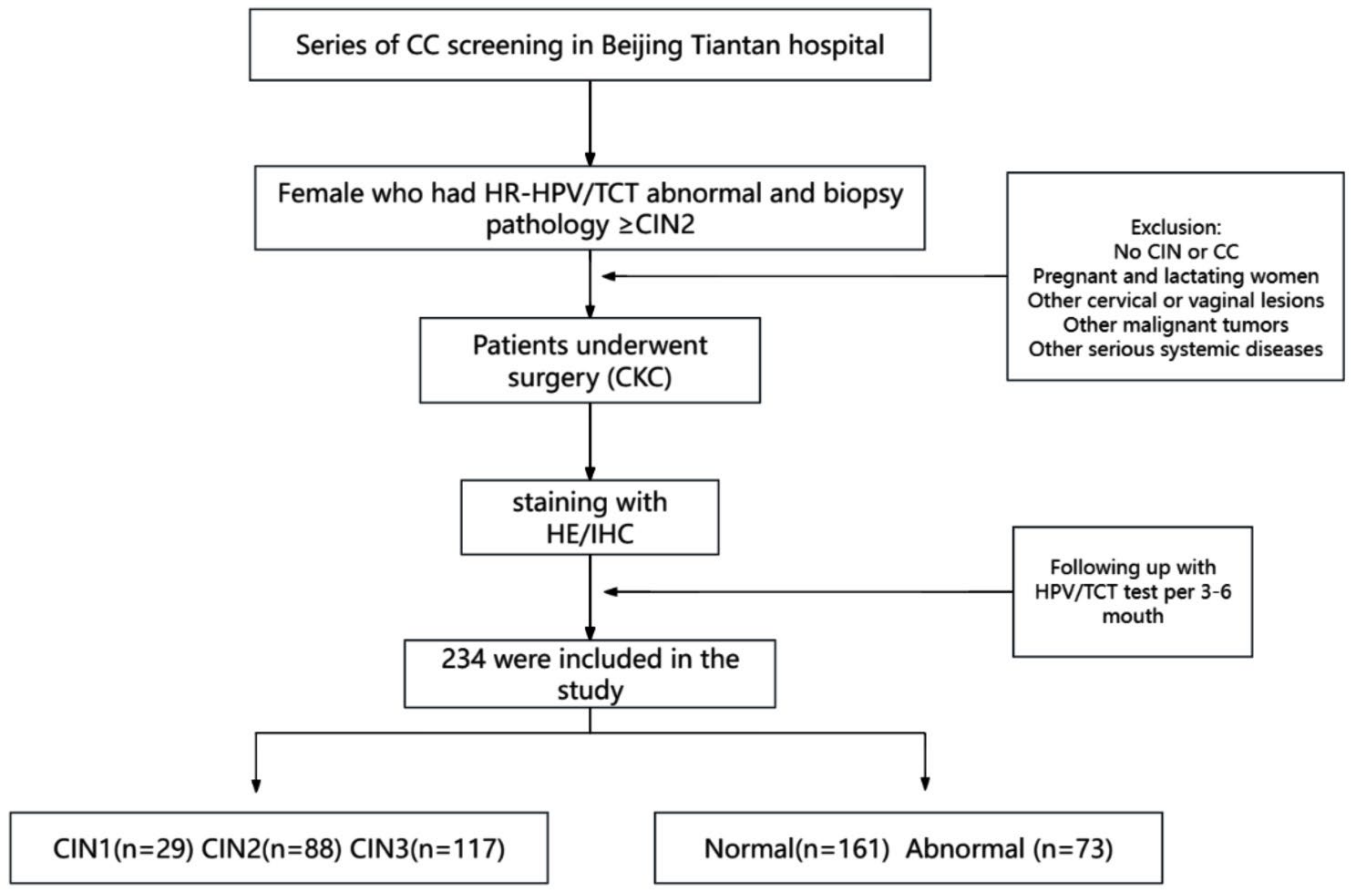
Flow chart of the study based on the screening cohort

Table 1 summarizes the baseline characteristics of the 234 participants in the study, with a mean age of 41 years and a standard deviation of 10. Regarding to·CIN grades, the majority of participants had CIN2/3 (87.6%). In terms of follow-up outcomes, 68.8% of participants had a normal response, while 31.2% of the participants had abnormal HPV/TCT test results within 1–2 years. Figure 1 shows the flow chart of the study based on the screening cohort with exclusions annotated.

**Table 1:** Patient demographics and baseline characteristics

Characteristic	*N* = 234^1^
**Age**	41 ± 10
**CIN**	
1	29 (12.4%)
2	88 (37.6%)
3	117 (50.0%)
**Eag1**	
0	15 (6.4%)
1	53 (22.6%)
2	95 (40.6%)
3	71 (30.3%)
**IS of p16/Ki-67**	
0–2	21 (9.0%)
3–4	135 (57.7%)
5–6	78 (33.3%)
**Follow-up of HPV/TCT**	
Normal	161 (68.8%)
Abnormal	73 (31.2%)

^1^Mean ± SD; n (%)

### Correlation analysis between the grade of CIN and Eag1 expression score, IS of p16/Ki-67

**Fig. 2:**
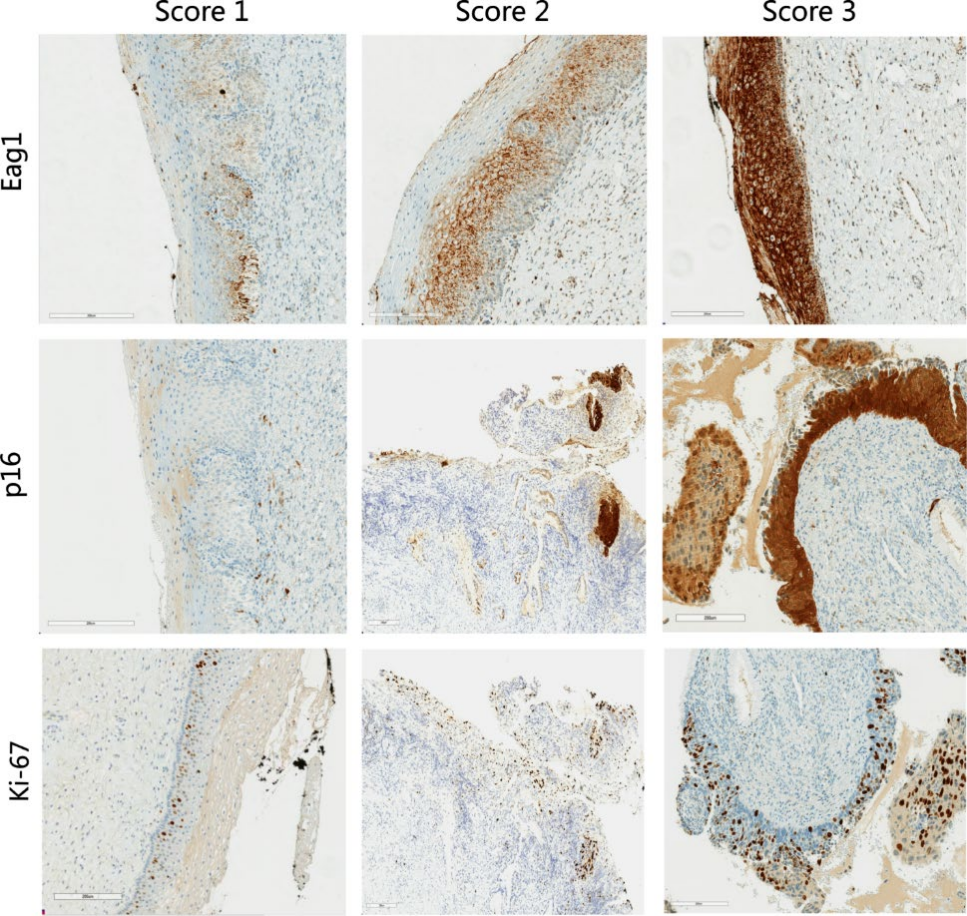
IHC score is correlated with CIN grade but not exactly corresponding. Eag1 staining corresponding Ki-67 and p16 stainings show positive predominantly found in the lower one-thirds of the epithelium (Score 1), which is most common in CIN1. Positive in the lower two-thirds of the epithelium (Score 2) is most common in CIN2. Positive in full-thickness of the epithelium (Score 2) is most common in CIN3+

The baseline characteristics table (Table 2) provides key insights into the distribution of the expression scores of Eag1 and IS of p16/Ki-67. Analysis of the data revealed that there were significant differences in the distribution of expression characteristic of p16/Ki-67 (*p* = 0.03) and Eag1 (*p* = 0.03) across the CIN groups. We focused on the expression of Eag1, which showed a similar correlation with CIN grade as IS score of p16/Ki-67 (Fig. 2). The half highest expression score 2/3 of Eag1 has a differences positive rate in the distribution in CIN1, CIN2 and CIN3 which were 62.1%, 69.3%, 74.4%, respectively. This suggests that the expression of Eag1 is related to the grade of CIN.

**Table 2:** Patient demographics and baseline characteristics

Characteristic	CIN			*p*-value
	1, *N* = 29	2, *N* = 88	3, *N* = 117	
**IS of p16/Ki-67**				0.03^1^
0–2	7	5	9	
3–4	15	49	71	
5–6	7	34	37	
**Eag1**				0.03^1^
0	6	5	4	
1	5	22	26	
2	9	39	47	
3	9	22	40	

^1^Fisher’s exact test

### Correlation analysis between follow-up results and CIN grade, Eag1 expression score, IS of p16/Ki-67

The follow-up data (Table 3) show the distribution of follow-up groups in relation to CIN, Eag1 expression score, and IS of p16/Ki-67. Among the 234 participants with CIN1-3, 73 cases had abnormal HPV/TCT test results at follow-up, and 161 cases stay normal until the end of the 2 years. For the Eag1 expression score, there was a significant difference in the distribution between the two follow-up groups (*p* < 0.001), and a higher score was associated with a greater proportion of abnormal cases. Similarly, for IS of p16/Ki-67, there were significant differences in distribution (*p* = 0.001). However, for the grade of CIN, the distribution did not show significant differences (*p* = 0.765), while the percentages in the abnormal group were similar in the CIN1-3 group (27.6%, 29.5%, 33.3%). It seems that the increased histological severity of CIN does not affect the follow-up outcomes after CKC.

**Table 3:** Patient demographics and follow-up data

Characteristic	Follow-up of HPV/TCT			
	Normal, *N* = 161	Abnormal, *N* = 73	Statistic	*p*-value
**CIN**			0.54	0.765^2^
1	21	8		
2	62	26		
3	78	39		
**Eag1**				< 0.001^3^
0	15	0		
1	49	4		
2	65	30		
3	32	39		
**IS of p16/Ki-67**			13.20	0.001^2^
0–2	18	3		
3–4	101	34		
5–6	42	36		

^2^Pearson’s Chi-squared test

^3^Fisher’s exact test

### Performance of the Eag1 expression score and IS of p16/Ki-67 to predict the follow-up result

**Fig. 3:**
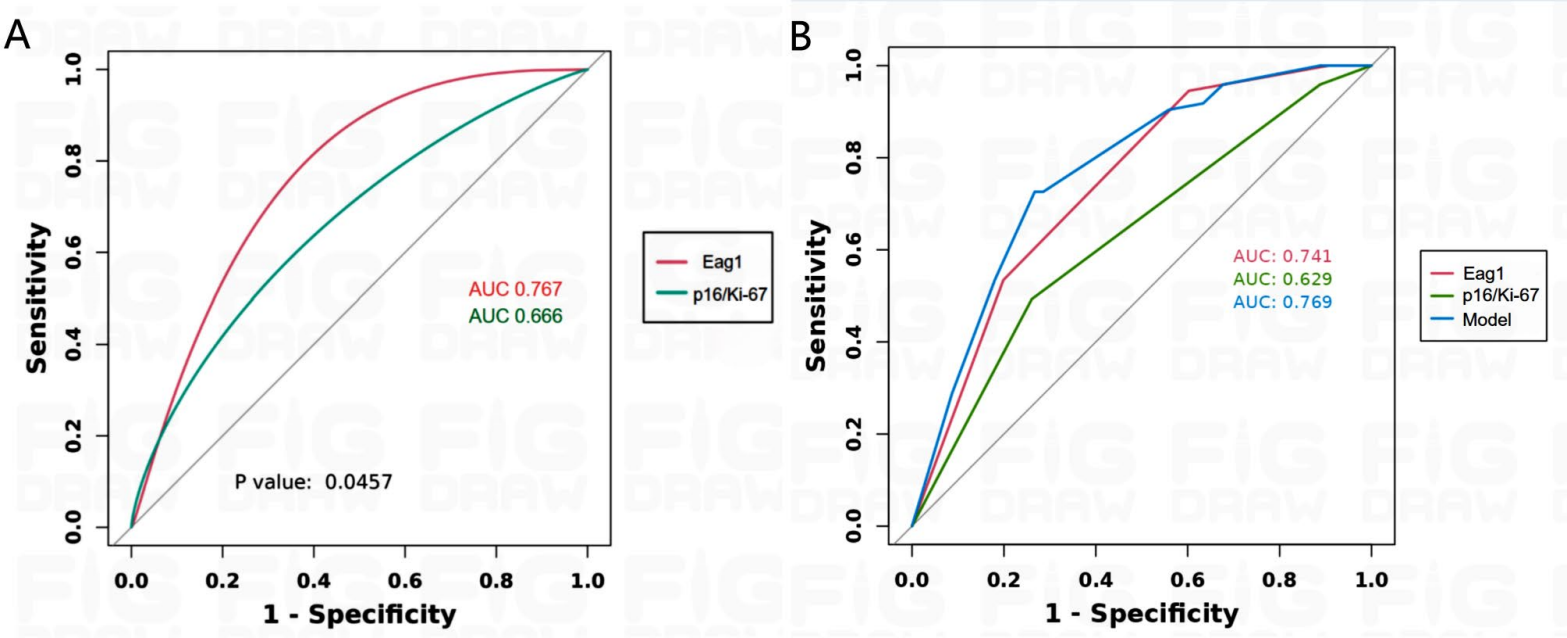
ROC curves of IHC score for detecting the results of following-up after CKC. (**A**) The Eag1 score showed advantages over IS of p16/Ki-67 with higher AUCs. (**B**)integrated indicator has highest AUCs than other indicators

To evaluate the possible efficiency of Eag1 expression score and IS of p16/Ki-67 as a strategy for predicting prognosis at follow-up, receiver operating characteristic (ROC) curves were generated based on the correlations between the results of the HPV/TCT tests at follow-up and the IHC expression scores of the biomarkers and making the curve smooth (Fig. 3A). The Eag1 score showed advantages over IS of p16/Ki-67 with higher AUCs (0.767 vs. 0.666). Based on the maximum Youden index (YI), the optimal cutoff points for each were chosen 2 for both. Using these cutoff points, the sensitivity (94.5% vs. 50.7%), specificity (79.8% vs. 76.1%), PPV (71.6% vs. 53.7%), and NPV (94.1% vs. 53.8%) was higher for Eag1 expression score than IS of p16/Ki-67.

Finally, in order to explore whether combined biomarkers can improve the predictive ability for CIN postoperative follow-up outcomes. we used Logistic regression analysis to combine biomarkers into a multi-marker and integrated the two scores into a sample model (Table 4), and then compared it with independent factors to obtain ROC curves (Fig. 3B). It showed that integrated indicator has advantages over Eag1 expression score or IS of p16/Ki-67 with higher AUCs (0.741 vs. 0.629 vs. 0.769).

**Table 4:** Logistic regression (model)

Characteristic	*N*	Event *N*	OR1	Estimate	SE1	95% CI1	*p*-value
**Eag1**	234	73	3.29	1.19	0.22	2.12, 5.10	< 0.001
**IS**	234	73	1.86	0.62	0.27	1.09, 3.18	0.024

^1^OR = Odds Ratio, ^1^SE = Standard Error, CI = Confidence Interval

Null deviance = 290; Null df = 233; Log-likelihood = -121; AIC = 247; BIC = 258; Deviance = 241; Residual df = 231; No. Obs. = 234

## Discussion

Grades of CIN reflects different factors that promote or accelerate the development of more advanced disease and factors that reduce or decelerate its progression. Although, in most cases it is possible to make a diagnosis by evaluating HE-staining sections, there are still some diagnostic difficulties, so appropriate biomarkers are needed to aid in the diagnosis [18–20]. The most widely and consistently used immunohistochemical stains in the cervix are p16 and Ki67, which are strongly and diffusely positive in most cases of CIN2-3. However, the specificity of p16 for CIN is limited by the different prevalence of carcinogenic HPVs across the entire spectrum of CIN1 [21]. Therefore, we realize that new indicators should be proposed and their ability to assist in diagnosis should be verified.

Eag1 is a protein for the selective transport of K^+^ [14]. We found a suspicious correlation between Eag1 expression and the malignancy degree of cervical cancer cells in a previous study [16]. It is similar to the response of p16/Ki-67 to cervical cancer cells, so we hope it could be used as an indicator of the biological characteristics of CIN.

In this study, IHC staining for Eag1 was performed and the correlation between Eag1 expression score and grades of CIN was evaluated. There was a positive correlation between them, and the expression of Eag1 increased with the severity of CIN, similar to the IS of p16/Ki-67. This suggests that Eag1 has the potential to improve the accuracy of the pathological diagnosis of CIN, even if there is no obvious advantage compared to p16/Ki-67 in terms of the strength of the correlation. The application value may provide a reference for the diagnosis of CIN in some sections with confusing HE staining.

However, we believe that the correlation between Eag1 expression score and grades of CIN has limited value when it comes to predict the progress of CIN. Actually, the purpose of CIN classification is to stratified the management of patients according to the different risk of progression, and less risk sometimes means expectant management [22]. According to the 2017 guidelines of ASCCP for the treatment of CIN, patients with a high risk of CIN2 + on colposcopy can undergo diagnostic resection immediately. This “see-and-treat” approach largely avoids the risk of CIN progressing to CC [23]. Therefore, for high risk patients of diagnostic resection, we should pay more attention to the risk of CIN persistence or recurrence after treatment. In fact, women with CIN 2 or CIN 3 retain an elevated risk of recurrence or even invasive cancer for years after treatment [24]. Thus, heightened surveillance has been a rule. However, our experience has shown that the rate of loss to follow-up after treatment for CIN is quite high, especially when patients have a false perception of a “complete cure” after cervical surgery. Confined by our limited medical resources, we would like to identify high-risk individuals with abnormal follow-up results and pay special attention to them.

A previous study evaluated the correlation between p16 and Ki67 expression levels in the conization of patients with HPV persistence/re-infection and CIN recurrence, indicating the clinical significance of p16 and Ki67 expression in guiding patient prognosis at follow-up [10]. Considering the expression of Eag1 showed a similar response to CIN as that of p16/Ki-67, we compared the distribution characteristics of the follow-up groups in relation to Eag1 expression score and IS of p16/Ki-67 in our study. Unlike previous studies, we set the end points of follow-up as the abnormal results of the HPV/TCT test. Because studies have proven the effectiveness and comprehensiveness of combined cytology and HPV testing in follow-up after CIN treatment [17], we believe that this combination can indicate a high risk of persistence or recurrence of CIN. We therefore broadened the population with risk in follow-up aimed to maximize screening sensitivity. Our study showed that both Eag1 expression score and IS of p16/Ki-67 were significantly different in the distribution between the two follow up groups. Moreover, Eag1 performed better in terms of correlation intensity. Notably, ROC curves of these biomarkers for detecting the results of follow-up indicated that Eag1 had better predictive potential than p16/Ki-67.

In particular, beyond our expectations, there was no significant difference in the follow-up results among the different grades of CIN. Additionally, we also observed that a part of patients in the CIN1 group still had persistent abnormal results of HPV/TCT test in their follow-up and that Eag1 was highly expressed in the tissue of cervix. Some experts deny that the HPV viral load is associated with the grades of cervical lesions, and they indicated that CIN1 was in the acute stage of HPV infection, and that the self-replication ability of HPV was significantly more prominent in other stages [25]. The HPV E6 E7 protein enhances Eag1 expression through the transcription factor E2F1 [16]. We estimated that higher Eag1 expression may predict more active of HPV and harder, which is more difficult to remove and contributes to abnormal tests during follow-up. This may be the reason why the patients in the CIN1 group did not show a better remission rate and may explain why the predictive potential of Eag1 was better than that of p16/Ki-67 in all CIN groups.

Finally, considering the similar predictive ability of Eag1 and p16/Ki-67 for predicting follow-up outcomes, we combined these biomarkers into a multimarker in order to explore whether it can improve the predictive ability than individual one. ROC curves showed that integrated indicators have higher AUCs over any apart which meaning that the simple model with two indices performed better at predicting follow-up outcomes. Due to the limited sample size, we could not confirm the predictive effect of these indicators in other CIN cohorts, but we confirmed that these indices had positive synergistic effects.

Nevertheless, our study has still some limitations. Firstly, IS of p16/Ki-67 system is based on the subjective assessment of distribution of IHC staining in CIN. Although this method increases the diagnosis of repeatability, it is still affected by subjective factors to some extent. Secondly, all patients in our study originated from only one medical center, and this was an observational cohort study; therefore, the data might not be adequate for a reliable conclusion. Furthermore, an abnormal HPV/TCT test does not fully represent the true risk of residual/recurrent disease of CIN after treatment, and the influencing factors may be complex. Accordingly, further multivariate analyses with larger sample sizes are needed.

## Conclusion

In summary, we confirmed a new protein, Eag1, that correlates with CIN grade and follow-up results after CKC. This may help us to improve the discernibility to risk populations after treatment for CIN.

## Data Availability

All data used in this manuscript is provided within the manuscript.
